# Oviposition Preference of Pea Weevil, *Bruchus pisorum* L. Among Host and Non-host Plants and its Implication for Pest Management

**DOI:** 10.3389/fpls.2015.01186

**Published:** 2016-01-06

**Authors:** Esayas Mendesil, Birgitta Rämert, Salla Marttila, Ylva Hillbur, Peter Anderson

**Affiliations:** ^1^Department of Plant Protection Biology, Swedish University of Agricultural SciencesAlnarp, Sweden; ^2^International Institute of Tropical AgricultureIbadan, Nigeria

**Keywords:** Bruchidae, host selection, insect behavior, legume, neoplasm, *Pisum sativum*, pea weevil

## Abstract

The pea weevil, *Bruchus pisorum* L. is a major insect pest of field pea, *Pisum sativum* L. worldwide and current control practices mainly depend on the use of chemical insecticides that can cause adverse effects on environment and human health. Insecticides are also unaffordable by many small-scale farmers in developing countries, which highlights the need for investigating plant resistance traits and to develop alternative pest management strategies. The aim of this study was to determine oviposition preference of pea weevil among *P. sativum* genotypes with different level of resistance (*Adet*, *32410-1* and *235899-1*) and the non-host leguminous plants wild pea (*Pisum fulvum* Sibth. et Sm.) and grass pea (*Lathyrus sativus* L.), in no-choice and dual-choice tests. Pod thickness and micromorphological traits of the pods were also examined. In the no-choice tests significantly more eggs were laid on the susceptible genotype *Adet* than on the other genotypes. Very few eggs were laid on *P*. *fulvum* and *L*. *sativus*. In the dual-choice experiments *Adet* was preferred by the females for oviposition. Furthermore, combinations of *Adet* with either *235899-1* or non-host plants significantly reduced the total number of eggs laid by the weevil in the dual-choice tests. Female pea weevils were also found to discriminate between host and non-host plants during oviposition. The neoplasm (*Np*) formation on *235899-1* pods was negatively correlated with oviposition by pea weevil. Pod wall thickness and trichomes might have influenced oviposition preference of the weevils. These results on oviposition behavior of the weevils can be used in developing alternative pest management strategies such as trap cropping using highly attractive genotype and intercropping with the non-host plants.

## Introduction

Field pea, *Pisum sativum* L. is a cool season legume crop grown in tropical highlands and in many countries in temperate regions (Messiaen et al., 2006). It is an important crop both for human consumption and for animal feed mainly due to its high protein content, and thus nutritional value. Furthermore, it provides ecosystem services by improving soil fertility through symbiotic nitrogen fixation (French, 2004; Khan and Croser, 2004; Messiaen et al., 2006). Insect pests are one of the major constraints of field pea production (Clement et al., 2000), among which the pea weevil, *Bruchus pisorum* L. is an economically important pest of field pea worldwide. In Ethiopia, seed damage and weight loss up to 85 and 59%, respectively, has been reported after attack by the pea weevil (Teka, 2002; Seyoum et al., 2012). As a consequence, the damaged seeds have low marketable value, are less valuable for human consumption and animal feed and show poor germination rate (Brindley et al., 1956; Clement et al., 2000, 2002; Seyoum et al., 2012).

The pea weevil has one generation per year and it reproduces only on field pea. Upon emergence from hibernation sites adult weevils fly into the pea fields and start to search for mate and oviposition sites. Egg laying starts about 2–2.5 weeks after the arrival of the weevils. The female weevil lays its eggs on pods of peas and upon hatching the first instar larva bore directly to the seed. Larvae develop inside the seed by consuming the content of the seed, which results in damage to the crop (Brindley et al., 1956). This cryptic larval feeding habit within the seeds makes it difficult both to monitor the infestation and to control the pea weevil with chemical insecticides. Thus, the most suitable time to control the pest would be before females commence oviposition (Horne and Bailey, 1991; Baker, 1998; Clement et al., 2000). Due to the long infestation period of adult weevils it has been reported that repeated chemical spraying is required to be effective (Baker, 1998). Furthermore, fumigation of harvested peas in the store can prevent further damage by pea weevil (Baker, 1998; Clement et al., 2000). However, insecticides are often unaffordable for small-scale farmers in developing countries such as in Ethiopia. Furthermore, insecticides can have adverse effects on human health and the environment. For example, recent studies showed improper use of insecticides among field pea growers in Ethiopia can expose the farmers to pesticide risks (Mendesil et al., 2016). Thus, development of alternative pest management strategies is needed.

Understanding of oviposition preference behavior in relation to host and non-host plants may provide useful information for developing alternative pest management strategies such as intercropping and trap cropping strategy for insect pest management (Shelton and Badenes-Perez, 2006; Cook et al., 2007; Finch and Collier, 2012). Intercropping is a traditional agronomic practice in Africa which has been shown to reduce pest damage (Abate et al., 2000; Smith and McSorley, 2000) and increase productivity of farm land (Vandermeer, 1989). A study conducted in Ethiopia showed that intercropping of maize, *Zea mays* L. with Ethiopian mustard, *Brassica carinata* A. Braun and potato, *Solanum tuberosum* L. reduced infestation by the stem borers, *Busseola fusca* Füller and *Chilo partellus* (Swinhoe) (Wale et al., 2007). A plant species or variety which is attractive to insect pest can also be planted as a trap crop to protect the main crop (Shelton and Nault, 2004; Shelton and Badenes-Perez, 2006). Trap cropping has been developed for control of various insect pests (a review of Shelton and Badenes-Perez, 2006) and there is an increasing interest in the use of trap crops for pest management.

In many herbivorous insects, understanding female choice of oviposition site is important for evaluating plant resistance and interaction between plants. Among a variety of plants, insect herbivores often show higher preference for particular host plant species, crop varieties and/or crop stages for feeding and oviposition (Bernays and Chapman, 1994). Thus, there can be large differences in plant attractiveness and resistance between different host plants and varieties of the same crop (Smith, 2005). Furthermore, various studies have shown that non-host plants can influence insect herbivore behavior in different ways such as disturbing host finding, masking of host plants and as an oviposition repellent (Vandermeer, 1989; Finch and Collier, 2012; Ratnadass et al., 2012). Most insect herbivores rely on morphological and chemical cues in location of oviposition sites and both morphological traits and secondary chemical metabolites play a crucial role in plant resistance against insect pest attack (Bernays and Chapman, 1994). Plant traits such as different types of glandular structures, wax layers and tissue thickness have been shown to influence oviposition behavior of insect herbivores (Bernays and Chapman, 1994).

In previous field experiments, we found variation in the susceptibility to pea weevil attack between different field pea genotypes, of which *Adet* genotype is highly susceptible to the weevil, and *235899-1* and *32410-1* are moderately resistant based on mean percent seed damage (Teshome et al., 2015). There are also studies showing that non-host plants also can affect host plant choice behaviors of the pea weevil (Annis and O’Keeffe, 1984b). Furthermore, a specific morphological trait reported in peas is the growth of neoplasm on the pod surface, a ‘postular-like outgrowth’ that is controlled by a single dominant gene, *Np* (Nuttall and Lyall, 1964). Oviposition of pea weevil on peas with *Np* gene has been found to result in development of neoplasm (Berdnikov et al., 1992; Doss et al., 2000). Interestingly, neoplasms are also formed when peas with *Np* gene are grown in the greenhouse under reduced UV wavelengths (Nuttall and Lyall, 1964; Snoad and Matthews, 1969). However, there is little information about how oviposition behavior in the pea weevil reflects resistance among genotypes and how it is affected by non-host plants. There is also no information if it is possible to take advantage of neoplasm as a resistant trait against pea weevil.

Although intercropping and trap cropping pest management methods have been used for major insect pests in various cropping systems elsewhere, e.g., control of *B*. *fusca* and *C*. *partellus* in maize and sorghum, *Sorghum bicolor* (L.) Moench in Africa (Khan et al., 2014), there is no available information on such management methods for pea weevil. Identifying host plants that are preferred to female pea weevil and those that are less or non-preferred, may pave the way to develop intercropping and trap cropping as pest management strategies for the pea weevil. Therefore, the aim of this study was to determine oviposition preference of pea weevil to field pea genotypes with different level of pea weevil resistance and to non-host plants. We also wanted to determine the influence of pod morphological traits and neoplasm formation on oviposition preference by pea weevil.

## Materials and Methods

### Plants

Three field pea, *P. sativum* L., genotypes were selected based on results obtained from field experiments conducted in northern and north-western Ethiopia during 2011–2012: Ebinat (12°10′ N 38° 05′ E), Liben (11° 50′N 37°10′ E), and Sekota (13° 00′ N 38° 50′E) (Teshome et al., 2015). The genotypes were: *Adet*, an improved variety which is highly susceptible to pea weevil, released by Adet Agricultural Research Center in Ethiopia in 1997, and *235899-1* and *32410-1* that both are gene bank accessions that were found moderately resistant (Teshome et al., 2015) obtained from the Ethiopian Institute of Biodiversity in Addis Ababa, Ethiopia. Furthermore, *235899-1* is a *Np* genotype with neoplasm formation in pods. Two non-host plants to pea weevil, wild pea, *Pisum fulvum* Sibth. et Sm. (*NGB 102148*) and grass pea, *Lathyrus sativus* L., were also included in the experiment. *P. fulvum* was obtained from the Nordic Genetic Resource Center (NordGen), Alnarp, Sweden whereas *L*. *sativus* was collected from Adet area, Ethiopia. Plants were grown to produce flowering branches and pods for the insect studies. All plants were grown in 2-L plastic pots with humus rich gardening soil (Weibulls, Sweden) in a biotron chamber (22°C, 75% RH, 12:12 h light/dark cycle) in 2014 at SLU, Alnarp.

### Insects

Field pea seeds infested with *B. pisorum* were obtained from harvest of field experiments conducted in Liben, north-western Ethiopia during 2011–2012 and from farmers’ seed stores in this area. Infested seeds were kept in a transparent plastic insect rearing cage (31 cm × 22.5 cm × 12 cm) at room temperature (20–24°C). Newly emerged adult pea weevils were used for the bioassays. The sex of the weevils was determined based on the presence of a small spine on the tibia of the middle leg of only male insects (Bousquet, 1990). Newly emerged weevils can be kept alive up to 1 year.

### Oviposition Bioassay

#### No-Choice Tests

A no-choice oviposition assay was conducted following the methods of Hardie and Clement (2001) and Clement et al. (2002) with some modification. A pair of male and female *B. pisorum* was introduced into a plastic insect rearing cage (31 cm × 22.5 cm × 12 cm) that was placed in a climate chamber (24°C, 60% RH, 12:12 h light/dark cycle). The weevils were provided a branch of a field pea plant with four to six fresh flowers every 2 days for 10 days before each experiment. In addition, weevils were provided distilled water and sugar solution on a cotton swab, which was changed every week. At the start of the experiment two flat pea pods, which is the preferred stages for oviposition by pea weevil (Hardie and Clement, 2001), were provided for oviposition. Each pod was placed hanging from the top of the cage using paper clip without damaging the pod, then a staples magnet (10 mm, Staples, Inc. Amsterdam, The Netherlands) was placed on the outer surface of the cage in order to fix/attach the pod. Only pods of one genotype per cage (*Adet*, *235899-1*, *32410-1*, *P*. *fulvum* and *L*. *sativus*) were provided to each weevil. Pods were changed daily and the number of eggs laid on each pod was counted under stereo microscope. For each experimental setup ten replications were made in a completely randomized design. The weevils were allowed to oviposit for 10 days.

#### Dual-Choice Tests

A similar experimental procedure as described above was followed in the dual-choice experiments, except that one pod of the control (*Adet*) and one pod from four test genotypes (*235899-1*, *32410-1*, *P*. *fulvum* and *L*. *sativus*) were placed in each cage.

#### Degree of Neoplasm Formation on Pods of *235899-1*

In order to determine the association of degree of neoplasm formation and number of eggs laid by the weevil, pods of *235899-1*, which were selected for no-choice oviposition bioassay were first assessed for degree of neoplasm before oviposition assays. The degree of neoplasm formation was determined into four classes (1–4; **Figure 3A**), where 1 = <25% of the pod surface is covered with neoplasm, 2 = 25–50% of the pod surface is covered with neoplasm, 3 = 50–75% of the pod surface is covered with neoplasm and 4 = >75% of the pod surface is covered with neoplasm. Then the level of neoplasm formation was correlated with (related to) the number of eggs laid per pod. Twenty pods were sampled for each class and in total eighty pods were sampled for this study.

### Morphological Traits of Test Genotypes

#### Pod Thickness

Thickness of pod wall was measured using an Absolute Digimatic Caliper (500-182-30, Mitutoyo, Japan). A measurement was done in the middle of both on the upper and bottom part of the pod. In total forty pods were measured per each test genotype (*Adet*, *235899-1*, *32410-1*, *P*. *fulvum* and *L*. *sativus*), where four fresh pods of flat stage were sampled from ten different plants.

#### Scanning Electron Microscopy

Scanning electron microscopy (SEM) was performed at SLU, Alnarp to examine if there were any differences on the pod anatomy of test genotypes. Fresh pods of flat stage were sampled from *Adet*, *32410-1*, *235899-1*, *P*. *fulvum* and *L*. *sativus*. Small pieces of the pods were fixed overnight at 4°C in a solution of 2.5% glutaraldehyde and 2% paraformaldehyde in 0,1 M Na-phosphate buffer, pH 7.2, dehydrated in a graded series of ethanol and critical-point dried (CPD 020, Balzers, Lichtenstein). Sample pieces were attached on stubs with double-sided tape external, internal or cros section surface of the pod upwards, and coated with a mixture of gold and palladium 3:2 in a sputter (JFC-1100, JOEL, Tokyo, Japan). Coated samples were examined with SEM (435VP, LEO Electron Microscopy Ltd., Cambridge, UK) with 10 kV.

### Statistical Analysis

No-choice oviposition and genotype combination data, and pod wall thickness was analyzed using one-way analysis of variance (ANOVA) using a generalized linear model. Mean oviposition were logarithmic transformed before analysis. For dual-choice test a Student’s *t*-test was used to analyze differences in oviposition on control vs. test genotypes. Spearman’s correlation analysis was used to determine the associations of number of eggs laid and level of neoplastic formation. All statistical analysis was done using MINITAB 16 statistical software.

## Results

### Oviposition Preference Test

#### No-Choice Tests

**FIGURE 1:**
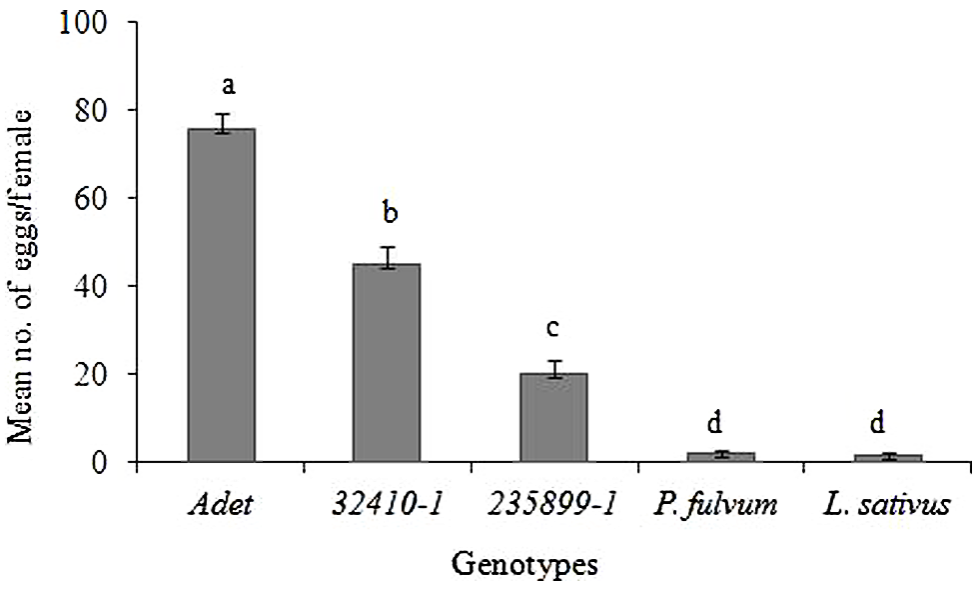
**Egg-laying activity (oviposition) of pea weevil females subjected to pods from *Pisum sativum*, *Pisum fulvum* and *Lathyrus sativus* genotypes in a no-choice test.** Bars marked with different letters are significantly different (Tukey’s test: *P* < 0.001). Bars indicate mean ± standard error (SE).

There were clear differences in the number of eggs laid on the different genotypes (*F* = 121.53; *df* = 4; *P* < 0.001, **Figure 1**). Female *B. pisorum* laid significantly more eggs on *Adet* genotype (an average of 76 eggs per female) than on the other test plants. On the other field pea genotypes, intermediate amounts of eggs were deposited, with fewer eggs on *235899-1* (*Np* genotype; an average of 20 eggs) than on *32410-1* (an average of 45 eggs). The two non-host leguminous plants *P*. *fulvum* and *L*. *sativus* received on average only 2 and 1.5 eggs, respectively.

#### Dual-Choice Tests

**FIGURE 2:**
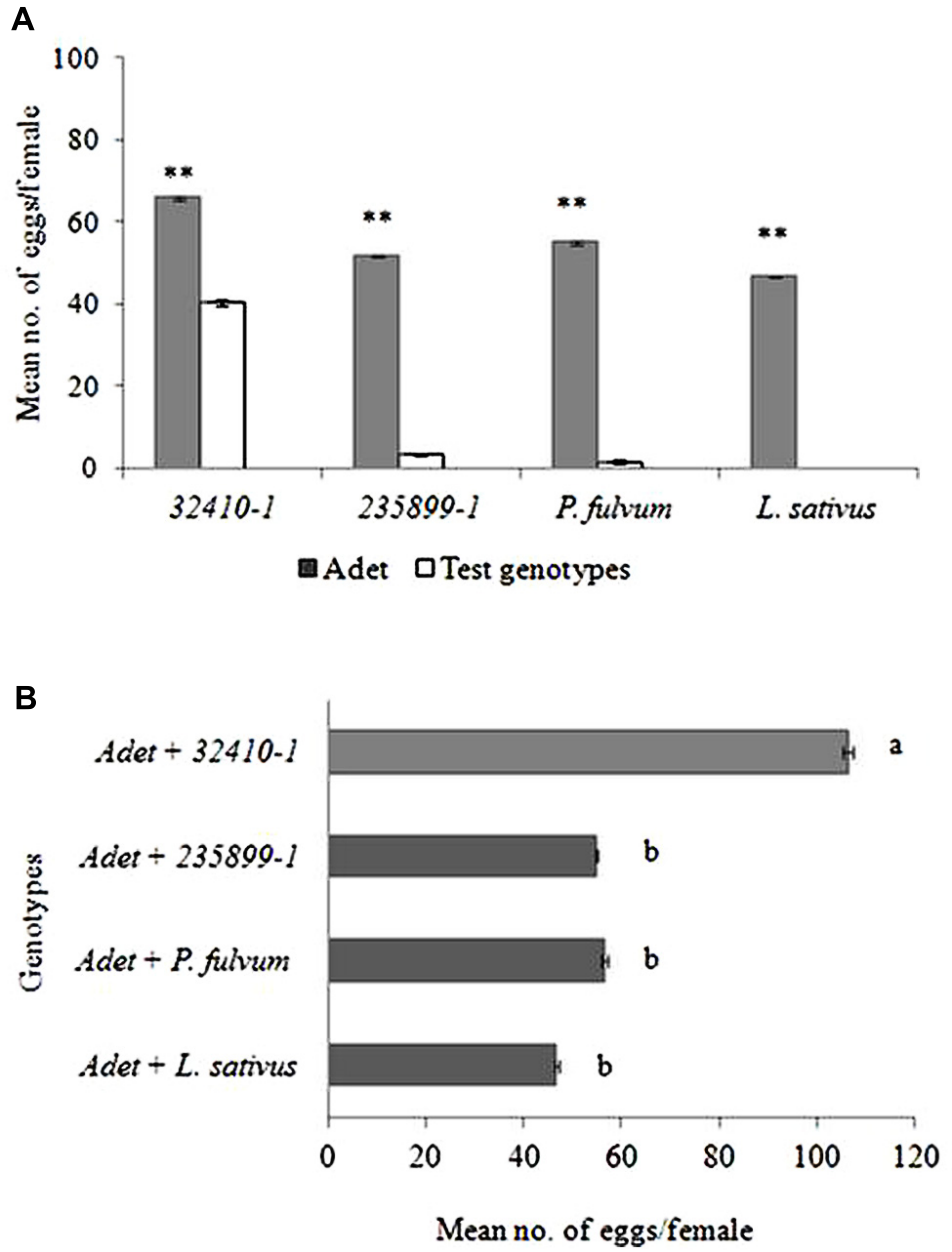
**(A)** Distribution of eggs of pea weevil in dual-choice oviposition tests. **(B)** Total number of eggs laid by the pea weevil in the experiments where the susceptible genotype *Adet* was combined with other more resistant pea genotypes and non-host plants. Bars marked with different letters are significantly different (Tukey’s test: *P* < 0.001; ^∗∗^*P* < 0.001). Bars indicate mean ± standard error (SE).

Given dual-choice *B. pisorum* females consistently laid significantly more eggs on *Adet* (control plant) (49–66 eggs per female) over the other plants tested (**Figure 2A**). With the exception of *32410-1* (40 eggs), (*T* = 4.66; *df* = 9; *P* < 0.005), the weevil laid very few number of eggs on the other test plants, i.e., *235899-1* (three eggs; *T* = 18.60; *df* = 9; *P* < 0.001), *P*. *fulvum* (two eggs; *T* = 16.55; *df* = 9; *P* < 0.001) and no oviposition was recorded on *L*. *sativus* (*T* = 7.68; *df* = 9; *P* < 0.001).

The total number of eggs laid varied depending on plant combination (*F* = 19.61; *df* = 3; *P* < 0.001, **Figure 2B**). The weevils laid significantly higher number of eggs when *Adet* (control plant) was combined with *32410-1* (106 eggs per female) than when *Adet* was combined with either *235899-1 (Np* genotype; 55 eggs) or non-host plants (49–57 eggs).

#### Degree of Neoplasm Formation on Pods of *235899-1* Compared to Oviposition

**FIGURE 3:**
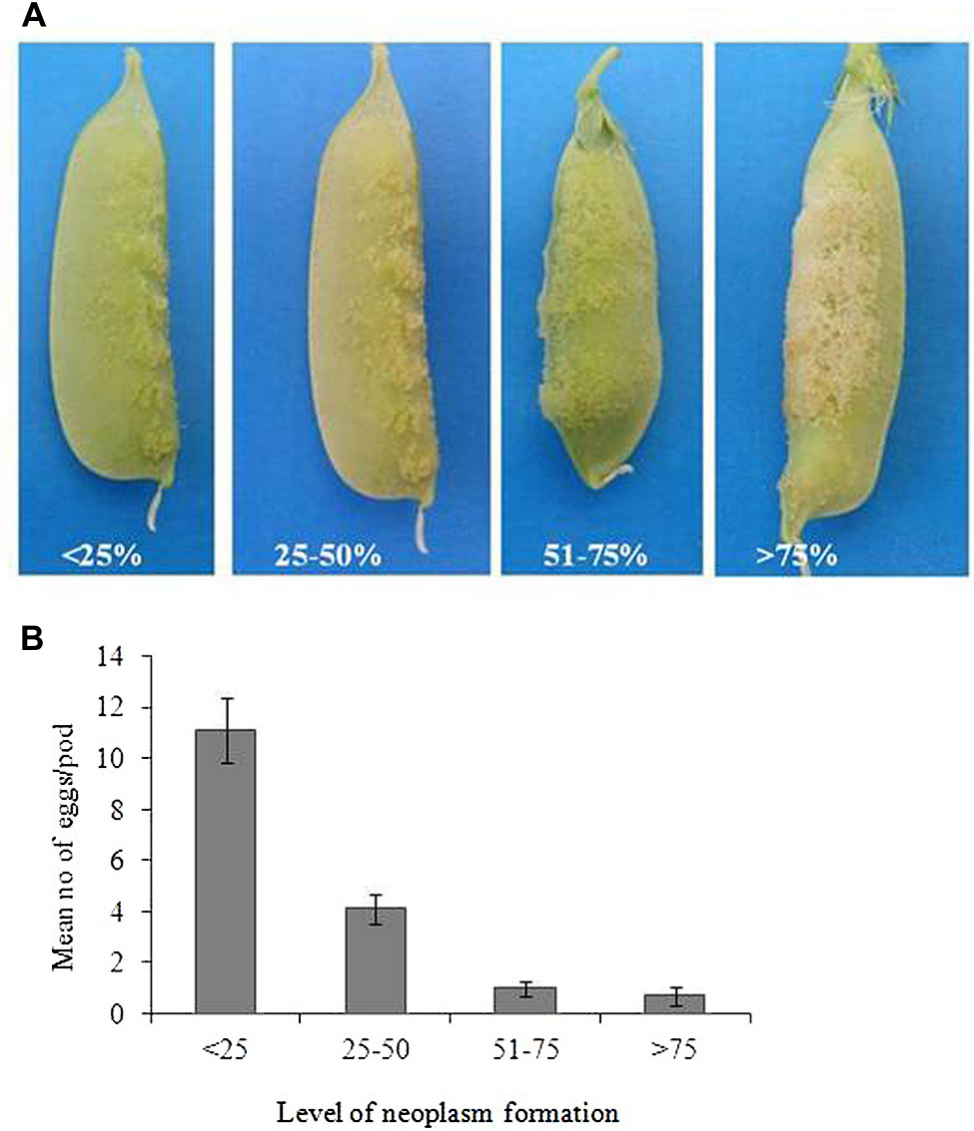
**(A)** Illustration of the degree of neoplasm formation on pods of *235899-1*. **(B)** Mean number of eggs laid on pods of *Np* genotype with different level of neoplasm formation. Bars indicate mean ± standard error (SE).

*Bruchus pisorum* females laid more eggs (11 eggs per pod) on the pods with <25% neoplasm formation followed by 25–50% (four eggs), while the weevil oviposited very few eggs (0.7–1) on pods with >51% neoplasm formation (**Figures 3A,B**). We found a negative correlation between the degree of neoplasm formation and number of eggs laid per pod (*r*_s_ = -1.0; *P* < 0.01).

### Morphological Traits of Pod

#### Pod Thickness

**FIGURE 4:**
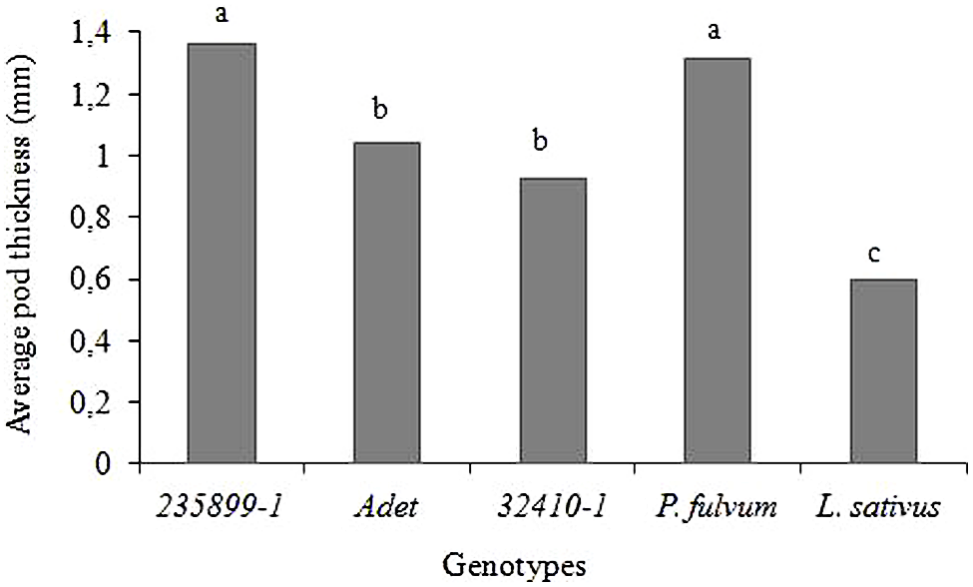
**Pod wall thickness of *P. sativum*, *P*. *fulvum* and *L. sativus* genotypes.** Bars marked with different letters are significantly different (Tukey’s test: *P* < 0.001).

The *Np* genotype *235899-1* and *P*. *fulvum* had significantly thicker pod wall with an average thickness of 1.36 and 1.31 mm, respectively, than *Adet* (1.04 mm) and *32410-1* (0.93 mm) (*F* = 103.86; *df* = 4; *P* < 0.001). *L. sativus* had an average pod wall thickness of 0.60 mm which was significantly lower than all other plants (**Figure 4**).

#### Scanning Electron Microscopy

**FIGURE 5:**
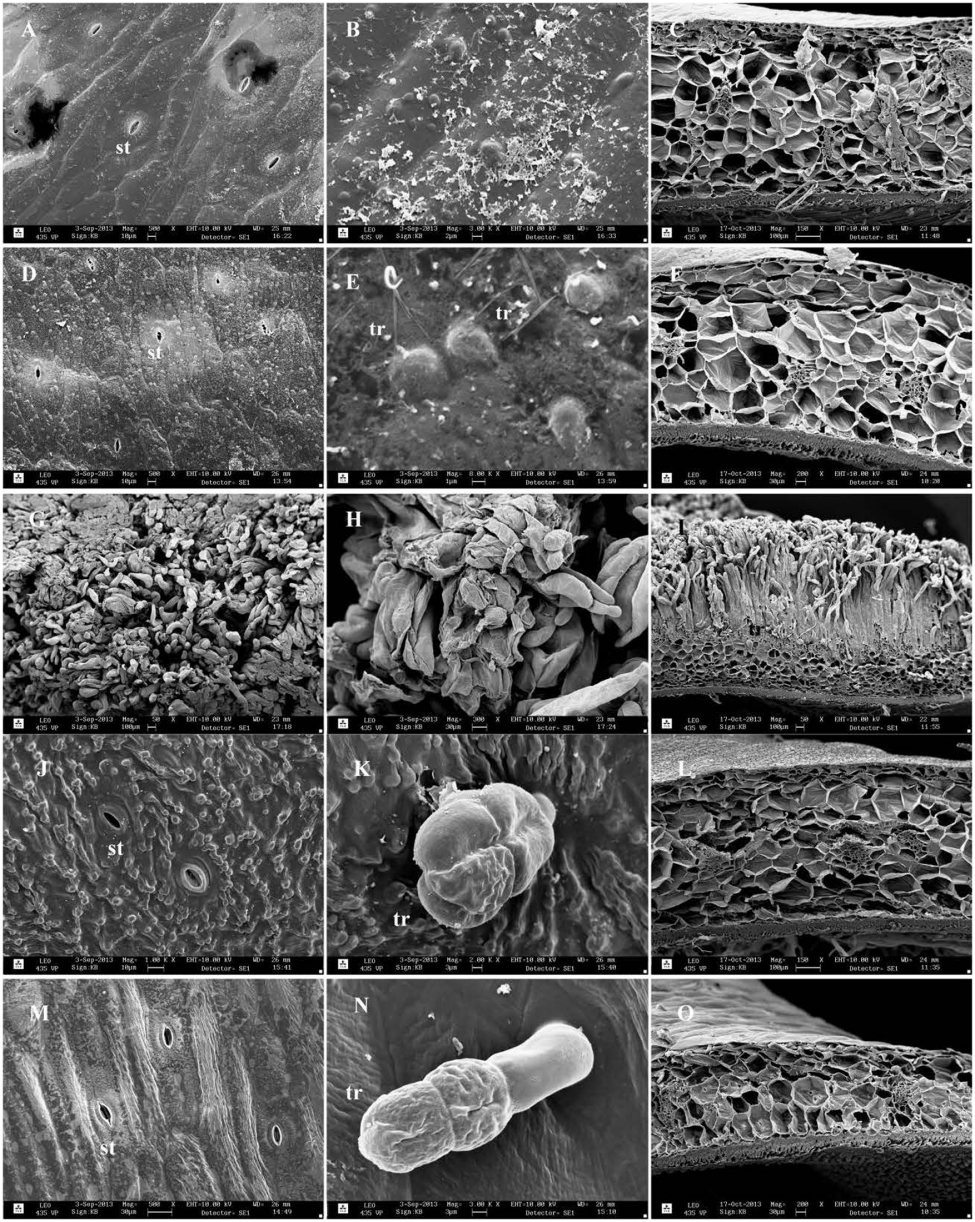
**Scanning electron micrographs of pod surface of field pea and non-host genotypes: **(A–C;***Adet*), **(D–F;***32410-1*), **(G–I;***235899-1*), (**J–L;***P*. *fulvum*), (**M–O;***L*. *sativus*).** The first and the second columns show external pod surface; the third column is cross section of the pod wall. st, stoma; tr, trichome.

Scanning electron micrographs of pods are shown in **Figure 5.** The external surface of the pods of *Adet* and *32410-1* as well as non-host plants was covered by a thick wax layer (**Figures 5A,D,J,M**). Different formations of epicuticular wax were observed, but as pointed out by Butler (2002), surface wax is unstable when exposed to changes in the environment, e.g., temperature and humidity. Occasional hairy trichomes were seen at least on *32410-1* surface (**Figure 5E**). Large glandular trichomes were only found on the pod surface of *P. fulvum* and *L. sativum* (**Figures 5K,N**). The external surface of the *Np* genotype *235899-1* was largely covered by an intensive neoplastic outgrowth seen as a mass of trichome-like filaments (**Figures 5G–I**) corresponding to the original description by Nuttall and Lyall (1964). Cros section of the pod with neoplastic outgrowth showed no clear epidermal cell layer, giving an impression that the neoplasm proliferation was directly from the parenchymal tissue. The neoplastic layer was almost twice as thick as the pod wall without the outgrowth. The thickness measurements of the pods (**Figure 4**) were in agreement with the size of the cros sections. It was evident that the thin pod of *L. sativum* had less parenchymal cell layers than *P. fulvum*, *Adet* and *32410-1*. There was variation in the number of cell layers of *235899-1* in the pod wall (not shown). Internal surfaces of the pods did not show any particular differences (not shown).

## Discussion

Our results show that female pea weevils discriminate between the tested field pea genotypes during oviposition. Both under no-choice and dual-choice tests, the weevils laid more eggs on *Adet* than on the other plants, indicating that *Adet* is highly preferred for oviposition. These results are supported by a field experiment that showed that *Adet* was highly susceptible to pea weevils and had over 90% seed damage (Teshome et al., 2015). Furthermore, we found that the genotypes *32410-1* and *235899-1* received intermediate amount of eggs, which is also in agreement with field results where those accessions showed moderate level of resistance to pea weevil. This indicates that oviposition behavior in the pea weevil reflects very well the resistance of different genotypes of field pea found in a field situation.

A potential explanation for the higher resistance to egg laying females found for the genotypes *32410-1* and *235899-1* can be that they are gene bank accessions, while *Adet* is an improved variety. It has been shown that domesticated crops and improved varieties which have been developed for specific traits, such as high yield and seed quality, might have lost their inherent resistance ability (Keneni et al., 2011; Tamiru et al., 2011; Chen et al., 2015). Furthermore, studies have shown that genetically uniform varieties are more likely to be prone to insect pest damage as compared to genotypically diverse cultivar mixtures (Tooker and Frank, 2012). Thus, as other improved crop varieties, it is conceivable that *Adet* may have lost its resistant ability as a consequence of breeding for other traits.

The present study also demonstrates that the pea weevil laid very few eggs on the non-host plants (*L*. *sativus* and *P*. *fulvum*) compared to the field pea genotypes tested. Furthermore, the combination of *Adet* with non-host plants resulted in lower total number of eggs laid by the weevils suggesting that the presence of non-host plants reduces oviposition by female weevils. This result corroborates earlier findings of reduced oviposition rate on *L*. *sativus* and *L. tingitanus* pods compared to *P. sativum* (Annis and O’Keeffe, 1984a,b) and of resistance in *P*. *fulvum* against the pea weevil (Clement et al., 2002). A recent review of Chen et al. (2015) also showed that gravid female insects in general prefer domesticated crops over their wild progenitors for oviposition. The reduced oviposition on the more resistant genotypes and on the non-host plants may depend on non-volatile chemical cues of the plants. It has been shown earlier that oviposition in pea weevil on *L*. *sativus* is probably attributed to deterrent compounds rather than absence of oviposition stimulants (Jermy and Szentesi, 1978; Annis and O’Keeffe, 1984b). Non-host plants commonly contain compounds that are deterrent for oviposition and feeding for most of insect herbivores (Bernays and Chapman, 1994). For example, in the Brassicaceae family glucosinolates act as a deterrent for generalist insects such as the green peach aphid, *Myzus persicae* (Sulzer), while they are feeding and oviposition stimulants for specialist insect species such as cabbage root fly,* Delia radicum* (L.) (Hopkins et al., 2009).

Plant morphological traits such as surface wax, trichomes, and the toughness of plant tissues can also play a crucial or at least partial role in plant defense against insect herbivores as physical or chemical barriers (Bernays and Chapman, 1994). Scanning electron microscopy (SEM) depicts occasional simple trichomes on the pod surface of *32410-1* and these might have contributed to a reduced oviposition on this genotype as compared to *Adet* that is without trichomes. In addition, the pod surfaces of *P*. *fulvum* and *L*. *sativus* showed glandular trichomes, which have also been reported on pods of other legume crops (Butler, 2002) and that have been shown to confer resistance against insect herbivores (Levin, 1973; Bernays and Chapman, 1994). Furthermore, *P*. *fulvum* had a thicker pod wall than the susceptible host genotype, which may partly have contributed to a lower oviposition rate by female weevils on this genotype. A morphologically based resistance mechanism in peas has been reported for pea weevil and pea leaf weevil, *Sitona lineatus* L., both causing a higher damage on pea plants with a reduced wax-layer (White and Eigenbrode, 2000; Chang et al., 2006). Similarly, in pigeon pea, *Cajanus cajan* (L.) Millsp. pod wall thickness and trichome density served as resistant traits to the pod fly, *Melanagromyza obtusa* (Malloch) (Moudgal et al., 2008) and in sorghum, leaf glossiness (due to wax) and trichome density were reported as important resistant traits to the sorghum shoot fly, *Atherigona soccata* Rondani (Chamarthi et al., 2011).

The genotype *235899-1* pods showed a neoplasm formation on the surface of the pods, but with variations in the degree of neoplasm formation between different pods (**Figure 3**). Pod anatomical features of *235899-1* showed an intensive neoplastic outgrowth on the external pod surface that is likely to form a physical barrier to gravid weevils. Consequently, we found a reduced oviposition on this genotype when expressing the neoplasm and the number of eggs laid was correlated to the degree of neoplasm formation on the pods. We also observed that during oviposition gravid female weevils wander on neoplastic pod surface and spend longer time before commencing oviposition than on pods of preferred genotypes. Interestingly, the pod surface with neoplastic outgrowth did not have a proper epidermal cell layer. Possibly the neoplastic outgrowth represents uncontrolled proliferation and differentiation of cells directly from the parenchymal cell layer. It has been discussed before whether the proliferation comes from hypodermis or epidermis (Nuttall and Lyall, 1964; Dodds and Matthews, 1966; Snoad and Matthews, 1969). The latter has also described the hair-like filaments at the later stages of neoplasm formation as seen in light microscope.

Variation in neoplasm formation might be related with the position of the pod on the plant and the stage of the pods (Nuttall and Lyall, 1964). In addition, the level of neoplasm may vary between different field pea accessions (Berdnikov et al., 1992). Neoplasm growth on field pea pod has mainly been reported in greenhouse grown peas with *Np* gene (Nuttall and Lyall, 1964; Snoad and Matthews, 1969; Burgess and Fleming, 1973), but there are also some reports about its formation on field grown peas under shade (Nuttall and Lyall, 1964; Doss et al., 1995), indicating the possiblity to induce the neoplasm formation in the field through intercropping with crops which can able to provide shade to peas.

Neoplastic growth has earlier been reported as a resistant trait against the pea weevil, which is expressed at the site of egg deposition due to induced plant defense response (Berdnikov et al., 1992; Doss et al., 1995, 2000). It reduces larval entry into the pod and may expose the eggs to biotic and abiotic mortality factors and hence minimizes infestation caused by the weevils (Berdnikov et al., 1992; Doss et al., 1995, 2000).

It may be possible to exploit oviposition preference of the pea weevil for the development of pest management methods such as intercropping and trap cropping. Non-host plants (*L*. *sativus* and *P*. *fulvum*), which are the least preferred plants by female weevils could be used as an intercrop to control pea weevil, but field studies are needed to assess the potential of these plants in reducing pea weevil infestation. Various studies have demonstrated the role of intercropping in reducing insect pest damage in different cropping systems. For example, Ali et al. (2007) reported that intercropping of field pea with *B. carinata* reduced pea aphid (*Acyrthosiphon pisum*) infestation and gave higher grain yield as compared to field pea planted as a monoculture. In addition, barley, *Hordeum vulgare* L., cultivars sown in a mixture has reduced bird cherry-oat aphid, *Rhopalosiphum padi* L., infestation compared to barley sown in pure stands (Ninkovic et al., 2002). Furthermore, a highly preferred host genotype can serve as a trap crop. Winter pea, *P*. *sativum*, for instance, showed a promising result as a potential trap crop to control pea leaf weevil in peas (Cárcamo and Vankosky, 2011). *Adet* is highly attractive for oviposition of pea weevil, which is one of the criteria to be a candidate trap crop (Shelton and Badenes-Perez, 2006). However, it also considerably supports larval development (Teshome et al., 2015) and thus has to be treated with conventional insecticide to make it a ‘dead-end-trap crop’ (Shelton and Nault, 2004; Shelton and Badenes-Perez, 2006). It would be interesting to test *Adet* in the field as a border trap and/or early planting to determine its potential under field condition.

## Conclusion

The present study demonstrates that female pea weevil discriminate between host genotypes and non-host plants during oviposition, *Adet* being highly attractive while the non-host plants were the least preferred by the females. The neoplasm formation attributed for a reduced oviposition rate on *235899-1*. Furthermore, thicknesses of pod wall and micromorphological traits appeared to influence oviposition behavior of gravid female weevils. Understanding oviposition behavior of the weevil among host and non-host plants may contribute to develop alternative pest management strategies such as trap cropping using a highly preferred genotype (*Adet*) and intercropping with the two non-host plants (*L*. *sativus* and *P*. *fulvum*). However, further studies are required under field conditions to test the preferred genotype for trap crop and the two non-host plants as an intercrops to reduce damage caused by the pea weevil.

## Author Contributions

EM, BR, YH, and PA conceived and designed the study. EM and SM collected the data and EM performed the analysis. BR and YH obtained the funding. EM wrote the manuscript with the assistance of PA, BR, YH, SM, and PA revised the manuscript.

## Conflict of Interest Statement

The authors declare that the research was conducted in the absence of any commercial or financial relationships that could be construed as a potential conflict of interest.
